# Development of Stabilizing Solution for Long-Term Storage of Bacteriophages at Room Temperature and Application to Control Foodborne Pathogens

**DOI:** 10.3390/v16071155

**Published:** 2024-07-17

**Authors:** Eo-Jin Kim, Min-Cheol Lim, Min-Ah Woo, Byoung Sik Kim, Jeong-A Lim

**Affiliations:** 1 Food Safety and Distribution Research Group, Korea Food Research Institute, Wanju-gun 55365, Republic of Korea; k.eojin@kfri.re.kr (E.-J.K.); mclim@kfri.re.kr (M.-C.L.); mawoo@kfri.re.kr (M.-A.W.); 2 Department of Food Science and Biotechnology, ELTEC College of Engineering, Ewha Womans University, Seoul 03760, Republic of Korea; b.kim@ewha.ac.kr

**Keywords:** bacteriophage, stabilizer, long-term storage, biocontrol, sanitizer

## Abstract

Bacteriophages (phages) have gained considerable attention as effective antimicrobial agents that infect and kill pathogenic bacteria. Based on this feature, phages have been increasingly used to achieve food safety. They are stored in a medium or buffer to ensure stability; however, they cannot be directly applied to food under these conditions due to reasons such as regulatory considerations and concerns about marketability. This study developed a stabilizing solution that allowed the maintenance of phage activity for extended periods at room temperature while being directly applicable to food. The stability of phages stored in distilled water was relatively low. However, adding a stabilizer composed of sugars and salts improved the survival rates of phages significantly, resulting in stability for up to 48 weeks at room temperature. When *Escherichia coli* O157:H7-contaminated vegetables were washed with tap water containing phages, the phages reduced the pathogenic *E. coli* count by over 90% compared with washing with tap water alone. Additionally, when pathogenic *E. coli*-contaminated vegetables were placed in a phage-coated container and exposed to water, the coating of the container dissolved, releasing phages and lysing the pathogenic *E. coli*. This led to a significant 90% reduction in pathogenic *E. coli* contamination compared to that after water rinsing. These results suggest an effective and economical method for maintaining phage activity and establishing the potential for commercialization through application in the food industry.

## 1. Introduction

Despite the development of food sanitation techniques, various foodborne pathogens still threaten human health, leading to hospitalization and even death [1]. Fresh food, particularly fruits and vegetables, is often implicated as the source of foodborne outbreaks, and virulent pathogens such as *Escherichia coli*, *Salmonella* spp., and *Listeria monocytogenes* are commonly found [2]. Fruits and vegetables are commonly grown in open environments, rendering them vulnerable to microbial contamination, and are typically not subjected to treatments to reduce or eliminate pathogens [3]. As ready-to-eat foods, agricultural products do not undergo further sterilization, making it crucial to address microbial loads through pre-treatment steps, such as washing, or post-treatment steps that involve using edible disinfectants [4].

Various methods have been developed to enhance the safety of fresh foods. Traditional approaches involve using natural or chemical food preservatives to control foodborne infections. However, the antibacterial activity of natural preservatives such as organic acids, bacteriocins, chitosan, and lactoferrin tends to be limited and relatively weak [5]. Chemical preservatives, while more effective, raise concerns regarding their potential side effects [6]. For example, chlorine-based sanitizers were commonly employed for washing vegetables to control microorganisms [7]. However, due to the formation of carcinogenic by-products and associated environmental and health risks, the use of chlorine is being reduced [8]. Consequently, researchers have been exploring alternative sanitizing agents to address these issues.

In recent years, bacteriophages (phages) have gained considerable attention as effective antimicrobials, overcoming the limitations of previous methods. Phages are viruses that infect and kill bacteria [9]. Lytic phages specifically target and propagate inside their host bacteria, ultimately leading to the lysis of the bacterial cell and the release of progeny phages [10]. The high specificity of phages ensures that they do not harm the normal and beneficial microbiota of the host, the food, or the environment [11]. Moreover, phages are not expected to have any detrimental effects on food quality or the health of animals and humans, eliminating the need for additional removal steps [12]. Due to these advantages, phages can be directly applied to food to control foodborne pathogens [13,14]. The approval of the first commercial phage product by the US Food and Drug Administration (FDA) as a “food additive” has led to the marketing of several phage-based products for the biocontrol of pathogenic bacteria, such as SalmoFresh™ (Intralytix Inc., Baltimore, MD, USA) for Salmonella control and EcoShield^®^ (Intralytix Inc., Baltimore, MD, USA) for *E. coli* control [15]. These products have been reported to control pathogens effectively and can be directly applied to food [16,17,18,19].

An essential aspect of developing and applying phage products lies in the strategy for phage storage and shelf life. Generally, phages are used in experiments in their state within the SM buffer. The SM buffer consists of NaCl, MgSO_4_, and Tris-HCL (pH 7.5), and gelatin is sometimes included as an optional component. Among these, NaCl and MgSO_4_ are approved as food additives. However, Tris-HCL is not approved as a food additive by FDA regulations [20], making it difficult to use in food, although the types of approved food additives may vary by country. Consequently, phages in their experimental state, included in the SM buffer, cannot be directly used in food. Actually, in commercial phage products such as PhageGuard Listex^TM^ (Micreos Food Safety, Wageningen, Netherlands), and PhageGuard S^TM^ (Micreos Food Safety, Wageningen, Netherlands) by Micreos, and SalmoFresh™ (Intralytix Inc., USA) and EcoShield^®^ (Intralytix Inc., USA) by Intralytix, aside from the phage components, most components have an existing regulatory status as regulated GRAS (Generally Recognized As Safe) ingredients or additives [21,22,23,24]. Additionally, even in the absence of regulatory issues, substances that could have a negative impact on the taste of food products, such as color, odor, and flavor, make it difficult to use them. Phages are well-preserved not only in SM buffer but also in liquid media; however, due to these reasons, their practical utility may be compromised.

When stored at room temperature, the stability of phages is compromised, and the most common strategy for phage preservation is storage at low temperatures, such as 4 °C, −20 °C, or −80 °C [25,26]. Phage products available in the market similar to the ones mentioned above are distributed in liquid form, and refrigeration (2−8 °C) in light-protected containers is recommended [21,22,23,24,27]. However, low-temperature storage has drawbacks, including associated energy costs and unsuitability for large volumes. Another method for phage storage is encapsulation, where phages are protected within a biomaterial [28], providing improved stability under storage and shipping conditions. Nevertheless, encapsulation does not enhance phage viability compared to low-temperature storage and introduces additional costs that must be considered based on the intended application [29]. Lyophilization, or freeze drying, is an effective preservation method that yields a dry powder that is easily stored and shipped. However, this process is complex, costly, and requires specific equipment [30].

In this study, we aimed to develop a stabilizing composition that could enable the long-term storage of phages at room temperature and be directly applied to food. We also assessed the antimicrobial activity of phages in controlling pathogen numbers in fresh foods. Based on these results, we propose a more convenient and cost-effective method for storing phage products and enhancing food safety.

## 2. Materials and Methods

### 2.1. Strains

*Escherichia coli* phage vB_EcoM_LEC1 (KCTC 15076BP, referred to as LEC1) and *Staphylococcus aureus* phage vB_SauH_LSA5 (KCTC 15395BP, referred to as LSA5) were isolated from sewage and a manure treatment plant in Iksan and Gimje, Jeollabuk-do, South Korea by this research group [31]. The pathogenic bacteria *E. coli* NCTC 12079 and *S*. *aureus* ATCC 25923 obtained from a bacterial culture collection were used as the host strains, respectively. Purified LEC1 and LSA5 phages were stored at 4 °C in tryptic soy broth (TSB, Difco, Detroit, MI, USA) composed of pancreatic digest of casein 17 g/L, papaic digest of soybean 3 g/L, dextrose 2.5 g/L, sodium chloride 5 g/L, and dipotassium phosphate 2.5 g/L. The phage titers were determined through spotting tests and the classic double agar overlay assay [32]. Briefly, the samples were serially diluted 10-fold with sodium chloride–magnesium sulfate (SM) buffer composed of 0.1 M NaCl, 8 mM MgSO_4_⋅7H_2_O, and 50 mM Tris-HCl, pH 7.5. Next, 10 μL of each dilution was spotted onto overlayed agar plates containing host bacteria or a mixture of 100 μL of each dilution, 100 μL of the host bacteria overnight culture, and 0.4% TSB soft agar was poured onto agar plates (*E. coli* NCTC 12079 for LEC1 and *S. aureus* ATCC 25923 for LSA5). The plaques were counted after an incubation at 37 °C for 8 (LEC1) or 16 h (LSA5). The detection limit for the phage plaque number is 10 PFUs/mL. In cases where plaques are not formed, ‘ND’ (not detected) is indicated on the bar graph, while on the line graph, it is represented as ‘1’, in accordance with the log scale.

### 2.2. Bactericidal Activity of Phages

An overnight culture of *E. coli* NCTC 12079 (10^9^ CFUs/mL) or *S*. *aureus* ATCC 25923 (10^9^ CFUs/mL) was inoculated into fresh TSB at a 1/100 volume ratio and shake-cultured at 37 °C. After approximately 2 h of incubation, when the culture reached the early exponential phase, 20 μL of phage LEC1 (10^9^ PFUs/mL) or LSA5 (10^8^ PFUs/mL) lysates was added with 2 mM CaCl_2_ to 180 μL of its target culture. The same volume of TSB was used as a negative control. During the incubation at 37 °C with shaking, the optical density at 600 nm was measured (SpectraMax; Molecular Devices).

An overnight culture of *E. coli* NCTC 12079, diluted 10,000-fold, was mixed with the phage stored in a stabilizing solution (see below) for 175 days at room temperature (3.6 × 10^6^ PFUs/mL) to an approximate multiplicity of infection (MOI) of 1. A 6 × 10^6^ PFUs/mL phage lysate concentration was used as a positive control. TSB was used as a negative control. After 2 h of shaking incubation at 37 °C, the mixtures were serially diluted in 10-fold increments, and 1 mL of them was spotted onto 3M Petrifilm, *E. coli*/Coliform Count Plates (EC). After a 24 h incubation at 37 °C, the numbers of colonies were counted.

### 2.3. Preservation of Phages in Different Suspended Solutions and Temperatures

To exchange the phage suspended solution, 2 mL each of LEC1 (10^9^ PFUs/mL) and LSA5 (10^8^ PFUs/mL) lysates were pipetted into Pierce Protein concentrators (100K MWCO) and then centrifuged at 6000× *g* for 30 min at 25 °C. The lysates were washed thrice by adding 20 mL of distilled water (DW) and centrifuged. The concentrated phage was resuspended in DW to a volume of 2 mL and then suspended in four different solutions. Four solutions were used for the phage suspension: SM buffer, saline (3M diluent, 0.85% sodium chloride), tap water, and DW. The number of plaques formed by active phages stored in each solution at room temperature or in the refrigerator was measured at different times.

### 2.4. Stabilizers

To add ions to the phage solution, NaCl and MgSO_4_⋅7H_2_O were dissolved in DW to final concentrations of 0.1 M and 8 mM, respectively. The solutions were then filtered through a 0.45 µm filter. D-Sorbitol (Sigma-Aldrich, St. Louis, MO, USA), D-(+)-maltose monohydrate (Sigma-Aldrich, St. Louis, MO, USA), and sucrose (Sigma-Aldrich, St. Louis, MO, USA) were dissolved in DW to final concentrations of 10% (*w*/*v*). After 1 h of mixing with a magnetic stirrer, the solutions were filtered (0.45 µm). The number of plaques of active phages stored in each solution at room temperature was measured according to the preservation time.

### 2.5. Coating Materials

Pullulan (Samyang, Daejeon, Republic of Korea), carboxymethyl cellulose (CMC; Sigma-Aldrich, St. Louis, MO, USA), polyvinyl alcohol (PVA; Sigma-Aldrich, St. Louis, MO, USA), corn starch (Sigma-Aldrich, St. Louis, MO, USA), and whey protein concentrate (WPC; Marquez Brothers International, San Jose, CA, USA) were used as candidates for the phage coating. The coating solutions with pullulan were prepared by dissolving 2.5 g of pullulan powder in a final volume of 50 mL of DW. In the case of CMC, 0.5 g of powder was added to DW, along with 0.5 g of glycerol and 0.125 g of Tween 80, reaching a final volume of 50 mL. PVA was dissolved in DW at a final concentration of 10% (*w*/*v*). Each material was individually mixed and stirred with heating using a magnetic stirrer. For corn starch, 7.5 g of the powder and 3 g of glycerol were dissolved to a final volume of 100 mL of DW and stirred with heating at 300 rpm for 30 min, followed by cooling before use. Dissolve 10 g of WPC powder in 50 g of DW by stirring for 30 min, add 30 g of 99% glycerol, and then fill with DW up to 100 g. The final concentration of WPC was 10%, and the weight ratio of glycerol to WPC was 3:1. The mixture was then placed in a 90 °C water bath, homogenized at 5000 rpm for 4 min, and cooled in an ice bath. After cooling, it was homogenized at 5000 rpm for 4 min and degassed using an ultrasonic bath for 30 min. A total of 500 μL of phage-containing coating solutions of LEC1 (10^9^ PFUs/mL) and LSA5 (10^8^ PFUs/mL) was dispensed into a 6-well microplate and stored in a desiccator to dry. For rehydration, 1 mL of SM buffer was added, followed by shaking for 15 min. The number of phage plaques was determined through a double-layered plaque assay or spotting assay.

To reconfirm the role of stabilizers in coated phages, a coating solution with 5% pullulan, 0.1 M NaCl, 8 mM MgSO_4_⋅7H_2_O, and 10% sorbitol was prepared using the described method. Phages LEC1 or LSA5 dispersed in distilled water (DW) were mixed with this coating solution. The resulting mixture was evenly distributed into 6-well microplates and allowed to air dry at room temperature for 24 h. Afterward, half of the plates were stored at refrigerated temperatures, while the other half remained at room temperature. On days 1, 3, 5, 7, 10, 14, 22, or 43, 1 mL of SM buffer was added to rehydrate the coating, and the plaque count of active phages was measured.

### 2.6. Application on Fresh Vegetables

#### 2.6.1. Vegetables Artificially Contaminated with Pathogenic Bacteria

Strawberries, kimchi cabbage, Brussels sprouts, and broccoli were purchased from local grocery stores in Jeonju, South Korea. They were washed with flowing tap water and dried naturally. One hundred microliters of *E. coli* NCTC 12079 culture (8 × 10^6^ CFUs/mL) was applied to the strawberries and kimchi cabbage and evenly spread across surfaces. Twenty microliters of the inoculum was loaded 10 times on the cut section of Brussels sprouts and broccoli. The *E. coli*-contaminated vegetables were left at room temperature for 60 min before the phage was applied.

#### 2.6.2. Phage Application: Direct Dropping

Phage LEC1 (6 × 10^10^ PFUs/mL), stored in DW containing 0.1 M NaCl, 8 mM MgSO_4_⋅7H_2_O, and 10% (*w*/*v*) sorbitol (hereafter referred to as stabilizer), was diluted 10- to 100-fold with a stabilizing solution (DW containing stabilizer) to achieve MOI values of 10,000, 1000, and 100. One hundred microliters of each solution was dropped on *E. coli*-contaminated strawberry and kimchi cabbage surfaces and spread across the surfaces. A stabilizing solution without phage was used as the negative control. After 30 min, the vegetables were transferred to sample bags and mixed with 100 mL of PBS, followed by stomaching, respectively. Then, the viable *E. coli* in PBS were serially diluted in 10-fold increments, and 1 mL of them was spotted onto 3M Petrifilm, *E. coli*/Coliform Count Plates (EC). After a 24 h incubation at 37 °C, the numbers of colonies were counted.

#### 2.6.3. Phage Application: Antibacterial Washing Solution

To make a vegetable washing solution containing phages, 5 mL of phage LEC1 (5 × 10^9^ PFUs/mL) stored in a stabilizing solution was added to 45 mL of tap water. *E. coli*-contaminated Brussels sprouts were inserted in the washing solution. Tap water mixed with a stabilizing solution without phage was used as the negative control. After 10 min, the number of viable *E. coli* remaining on vegetables was counted using EC petrifilm with the method described in Section 2.6.2.

#### 2.6.4. Phage Application: Convenient Phage-Coated Rinsing Containers

Two milliliters of a 5% pullulan solution containing the stabilizer and phage LEC1 (10^10^ PFUs/mL) were poured into a plastic container, spread widely, and then air dried at room temperature for 24 h. Pathogenic *E. coli*-contaminated Brussels sprouts or broccoli were placed inside the container, and 20 mL of tap water was added, followed by shaking. After 10 min, the vegetables were transferred from the plastic container to a sterile bag and mixed with 50 mL of PBS, followed by stomaching. Then, the viable *E. coli* in PBS were serially diluted in 10-fold increments, and 1 mL of them was spotted onto EC petrifilm. After a 24 h incubation at 37 °C, the numbers of colonies were counted.

## 3. Results

### 3.1. Effects of the Phage Suspended Solution and Temperature on Phage Stability during Preservation

**Figure 1:**
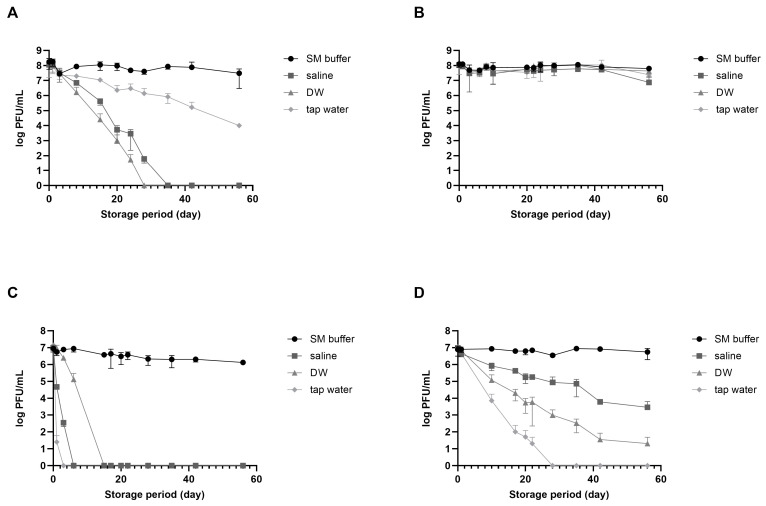
Phage stability in different solutions suitable for food consumption. Two types of phages were dispersed in sodium chloride–magnesium sulfate (SM) buffer (circle), saline (rectangular), distilled water (DW; triangle), or tap water (diamond) and preserved at either room temperature or in the refrigerator. Analyses of phage plaque counts were carried out periodically on representative samples. (**A**) *E. coli* phage LEC1, room temperature; (**B**) *E. coli* phage LEC1, refrigerator; (**C**) *S. aureus* phage LSA5, room temperature; and (**D**) *S. aureus* phage LSA5, refrigerator. Values represent the means with the standard deviations of three trials.

vB_EcoM_LEC1 (referred to as LEC1) is a lytic phage isolated using pathogenic *E. coli* as the host and belongs to the *Straboviridae* family, genus *Mosigvirus* [31]. vB_SauH_LSA5 (referred to as LSA5) is a lytic phage isolated using *S*. *aureus* as the host and belongs to the *Herelleviridae* family, genus *Kayvirus* (Figure S1). We chose two phages as model systems to develop a stabilizing agent for long-term phage storage at room temperature, focusing on their high activity and different stabilities. Phages LEC1 and LSA5 exhibited strong antimicrobial activity against pathogenic *E. coli* O157:H7 and *S*. *aureus*, respectively (Figure S2). To investigate the effects of suspended solutions and temperature on phage stability, we used three representative solutions appropriate for application on food (saline, DW, and tap water) and SM buffer as positive control. When stored at room temperature for eight weeks, *E. coli* phage LEC1 stability was maintained only in SM buffer, while it decreased in the other three solutions (Figure 1A). The phage titers in saline or DW tended to decrease. Eventually, the phages completely lost their activity before 30 days were completed. The titers of phages stored in tap water gradually decreased, although the activity remained relatively high. In contrast, when stored at 4 °C, the number of active phages remained consistent, regardless of the type of suspended solution, and their activity did not decrease considerably even after 56 days (Figure 1B). *S*. *aureus* phage LSA5 exhibited relatively lower stability under most conditions. When stored at room temperature, this phage lost all activity within 3 days in tap water, 7 days in saline, and 14 days in DW (Figure 1C). At 4 °C, the phages were more active than those stored at room temperature but showed a tendency for decreased activity, except when stored in SM buffer (Figure 1D).

The differences in stability observed for the different solutions cannot be generalized to all *E. coli* and *S*. *aureus* phages due to the limited use of only two specific phages in the experiments. Nevertheless, the activity of phages stored in solutions suitable for food applications decreased with increasing storage time. Notably, phage activity may still decrease even after storage at refrigerated temperatures. Consequently, alternative technologies must be developed for long-term storage, especially at room temperature.

### 3.2. Development of a Stabilizer for Long-Term Storage

Considering that phages dispersed in SM buffer exhibited long-term stability, the role of the ions in the SM buffer in enhancing phage stability was evaluated. When phages were stored in DW, the number of active phages decreased, and finally, all activity was lost within 40 (LEC1) or 20 (LSA5) days of storage (Figure S3). On the other hand, when NaCl and MgSO_4_ were added at the same concentration as in the SM buffer, a significant number of active phages remained for up to 63 days. These results demonstrated that ions such as NaCl and MgSO_4_ act as stabilizers that allow for the retaining of good residual titers and phage activity even after more than 2 months of storage (Figure S3).

The possibility of using saccharides as stabilizers was examined to reduce the slight decrease in phage activity even when ions were added. The role of saccharides varied, depending on the type of saccharide and phage (Figure S4). LEC1 was more stable in the presence of sorbitol or sucrose than in the negative control; no significant difference in the effect of the two sugars was noted, although sorbitol was slightly more effective. In the case of LSA5, adding sorbitol and sucrose initially increased stability. However, over time, phage plaques could only be detected in sorbitol-containing samples. In contrast, the addition of maltose inactivated both phages faster than treatment with control. These results suggest that, among the sugar additives tested, sorbitol is most effective for preserving the activity of phages stored in an aqueous solution. The effect of sorbitol as a stabilizer was maintained in both phages throughout the 63-day observation period (Figure S4).

**Figure 2:**
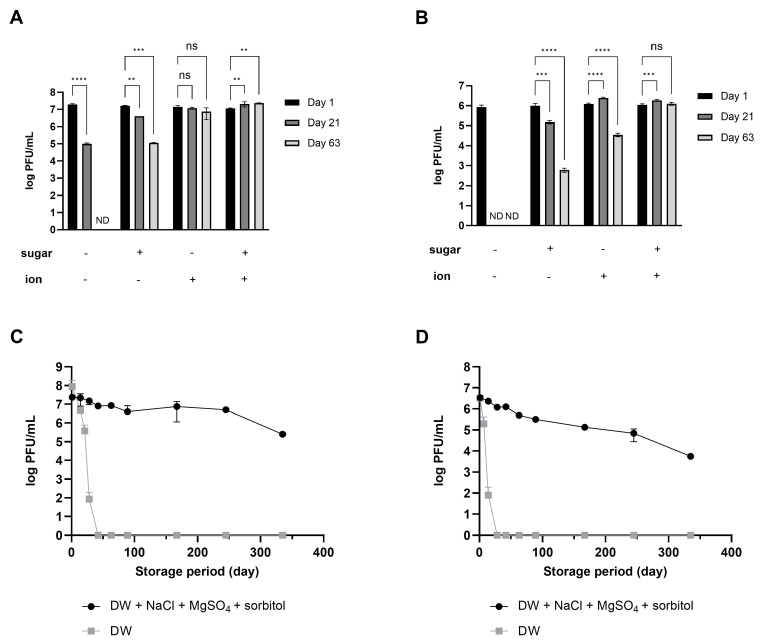
Synergistic effect of ions and sugar on phage stability during long-term storage. The numbers of phage LEC1 (**A**) and LSA5 (**B**) suspended in DW with or without 10% sorbitol or 0.1 M NaCl and 8 mM MgSO_4_ were measured at 1, 21, or 63 days of storage. ND; not detected (limit of quantification; 10 PFUs/mL). The plaque numbers of phage LEC1 (**C**) and LSA5 (**D**) stored in DW with 10% sorbitol, 0.1 M NaCl, and 8 mM MgSO_4_ were monitored for up to 48 weeks of storage at room temperature. Values represent the means with standard deviations of three trials. (ns, not significant; **, *p* < 0.01; ***, *p* < 0.001; ****, *p* < 0.0001).

Using salts (NaCl and MgSO_4_) for both phages was relatively more effective than sorbitol (Figure 2A,B). Importantly, the combined effect was overwhelmingly high, given that activity was maintained for up to 48 weeks of storage at room temperature despite a slight decrease (Figure 2C,D). Phage products marketed as food additives have a shelf life set between 6 months and 1 year, even when stored under refrigeration [23,24]. The fact that phages stored at room temperature maintained activity for a similar duration serves as evidence that the stabilizer developed in this study has enhanced the stability of phages. Based on these results, the composition and concentration for enhancing phage stability were optimized to include 10% sorbitol, 0.1 M NaCl, and 8 mM MgSO_4_⋅7H_2_O. This combination will hereafter be referred to as the ‘stabilizer’.

### 3.3. Bactericidal Activity of Long-Term Preserved Phage

The growth of *E. coli* treated with the phages was compared to assess whether the phages stored in the stabilizer showed the same bactericidal activity as the phage lysate stored in a medium in the refrigerator at the same concentration. After 2 h of incubation, the number of bacterial cells treated with phage lysate decreased by 4.17 logs (4.2 × 10^6^ CFUs/mL to 2.8 × 10^2^ CFUs/mL), while treatment with phage stored for a long time (175 days) with stabilizers reduced the number of bacteria by 4.04 logs (4.2 × 10^6^ CFUs/mL to 3.8 × 10^2^ CFUs/mL) (Figure S5). Considering that the titer of the phage lysate used in this experiment was slightly higher, the bacterial reduction rate can be interpreted as similar for both treatments. Consequently, the phages dispersed in the stabilizing solution maintained their activity for a long period and showed quantitatively equivalent antibacterial ability.

### 3.4. Efficacy of Direct Phage Application of Phage on E. coli-Contaminated Fresh Vegetables

**Figure 3:**
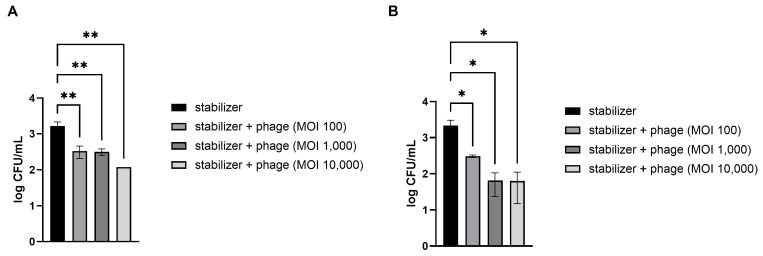
Efficacy of the phage solution in reducing *E. coli* O157:H7 levels in vegetables. Different concentrations of phage solution were directly dropped on *E. coli*-contaminated strawberries (**A**) and kimchi cabbage (**B**). After 30 min, the numbers of remnant *E. coli* on the vegetables were counted. MOI (multiplicity of infection) represents the ratio of phage to bacterial quantities. Values represent the means with standard deviations of three trials. The marks above the bars, * or **, represent significant differences at *p* < 0.05 or *p* < 0.01, respectively.

Fresh strawberries and kimchi cabbage were artificially contaminated with pathogenic *E. coli*. The preserved LEC1 phage was directly applied without other treatments because all materials, including the dispersed solution and stabilizers used in this study, are suitable for food application. The results demonstrated that treatment with the phages significantly reduced *E. coli* levels in a dose-dependent manner (Figure 3). Compared to the control, which contained only a stabilizing solution without phages, mean reductions of 72, 83, and 94% of pathogenic *E. coli* numbers in strawberries were obtained following phage treatment at MOIs of 100, 1000, and 10,000, respectively (Figure 3A). The phage solution was also effective at reducing the levels of *E. coli* on the kimchi cabbage surface at all concentrations examined (Figure 3B). Among the different doses, the lowest dosage (MOI of 100) resulted in an 81% reduction in *E. coli* numbers compared to that after application of the control and 96% and 98% reductions after application of the next doses (MOIs of 1000 and 10,000, respectively).

### 3.5. Efficacy of Phages in a Washing Solution on E. coli-Contaminated Fresh Vegetables

**Figure 4:**
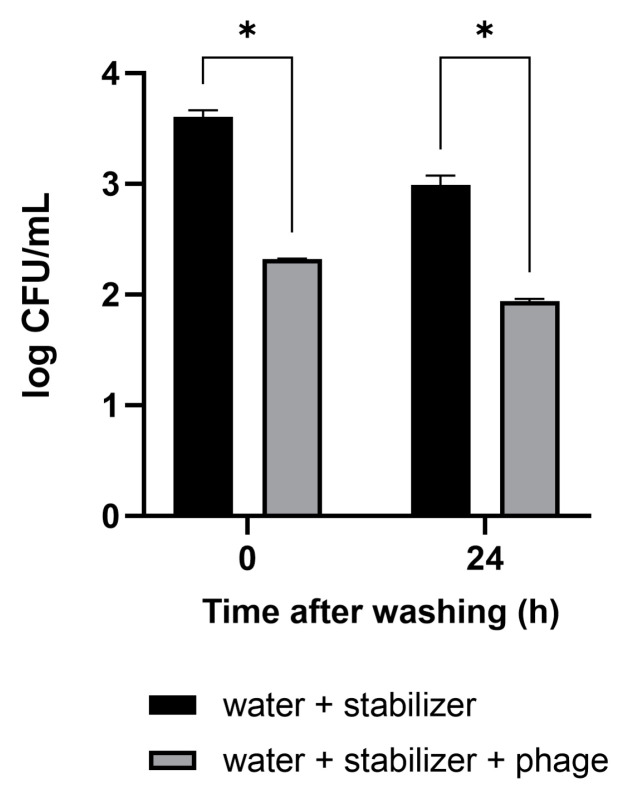
Efficacy of the phage solution as a washing material for the reduction of *E. coli* O157:H7 levels in vegetables. Brussels sprouts contaminated with pathogenic *E. coli* were washed with tap water containing the phage solution. The numbers of the remnant *E. coli* were counted immediately after washing and 24 h after refrigerated storage following the wash. The antibacterial activity of the phage solution is shown in comparison to tap water as a negative control. Values represent the means with standard deviations of three trials. * Significant at *p* < 0.05.

The antibacterial activity and convenience of use of the phage solution developed in this study were confirmed in vegetables that are difficult to wash. The cut sections of the Brussels sprouts were artificially contaminated with pathogenic *E. coli*. The vegetables were washed by immersion in a sample cup containing tap water with the phage solution for 10 min. Tap water-containing stabilizer without phage was used as a negative control. After washing, 3.0 × 10^3^ CFUs/mL of *E. coli* remained on the Brussels sprouts in the control condition (Figure 4). The concentration of *E. coli* in Brussels sprouts washed with the mixture of phage–tap water was measured to be 2.1 × 10^2^ CFUs/mL, 93% lower than that in the control condition. In Brussels sprouts refrigerated after the wash, the antibacterial activity was sustained even after 24 h.

### 3.6. Creation of Convenient Phage-Coated Rinsing Containers

To enhance the practical application of the phage’s antibacterial properties, we developed a convenient rinsing container coated with phages based on these results. The process involved placing vegetables inside a container coated with phages. The coating dissolves when water is added to the container, releasing the phages. The released phages control the level of pathogenic bacteria on the vegetables, ensuring effective disinfection and safety.

**Figure 5:**
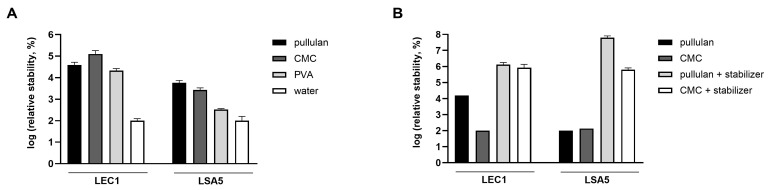
Stability of hydrated phages. Phages were coated with pullulan, carboxymethyl cellulose (CMC), or polyvinyl alcohol (PVA), and the number of plaques of the rehydrated phages were compared (**A**). The plaque number under the water-dried condition without the coating (negative control) was set to 100 for each phage, and the relative values were represented accordingly. For pullulan and CMC, the effect of stabilizer addition was also assessed (**B**). After applying a coating for 5 days, the number of plaques of the hydrated phages was counted. The relative values were obtained after the minimum value per phage was normalized to 100. Values represent the means with standard deviations of three trials.

For this purpose, the coating material needs to have the ability to maintain phage activity, dissolve easily in water, and be suitable for washing vegetables. In contrast to WPC and corn starch, which formed a highly turbid film and remained as small grains after rehydration, pullulan, CMC, and PVA formed transparent and even films. Phages were coated with these substances, and the stability of the hydrated phages was compared in the following order. The concentration of phages was measured for each coating material, and the change in the phage plaque count before and after coating was calculated (by dividing the plaque count after drying by the plaque count before drying). To compare the effects of each coating material on LEC1 and LSA5, which have different absolute values, the relative quantities were calculated based on each phage being coated with tap water as a reference. As a results, phages dried with any coating material were more stable than those dried with water (Figure 5A). Furthermore, it was demonstrated that the developed stabilizer significantly enhanced the stability of phages, not only in the solution state but also when dried.

**Figure 6:**
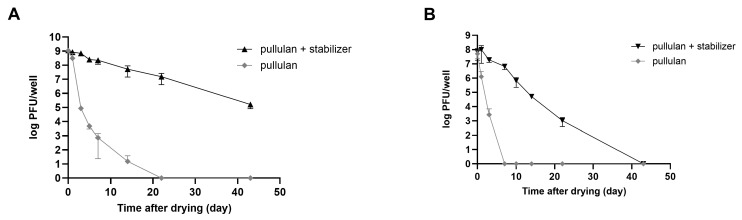
Stability of coated phages during storage at room temperature. Phages LEC1 (**A**) and LSA5 (**B**) were mixed with pullulan with or without stabilizer and applied as a coating on 6-well microplates. After drying, the plaque numbers of rehydrated phages were measured as a function of the extended storage time. Values represent the means with standard deviations of three trials.

Consequently, a substantial number of plaques was observed in rehydrated phages that were coated with the stabilizer. The relative stability of both phages was highest for the pullulan and stabilizer coatings (Figure 5B). In the case of LEC1 phage coated with only pullulan, the number of phage plaques dropped about 6 logs (1.1 × 10^9^ PFUs/mL to 7.4 × 10^2^ PFUs/mL) after 7 days of storage. Finally, there was no active phage after 14 days. However, when the stabilizer was included, the phage titers remained similar to the initial values after 7 days (1.2 × 10^9^ PFUs/mL to 2.3 × 10^8^ PFUs/mL), and considerable activity remained after 43 days of storage (Figure 6A). Even for the relatively less stable LSA5 phage, we confirmed stability for up to 7 days after coating (6.8 × 10^7^ PFUs/mL to 6.6 × 10^6^ PFUs/mL) under the optimal conditions, and considerable activity was exhibited for an extended period thereafter (Figure 6B). When stored at refrigerated temperatures in the presence of stabilizers, both phages maintained high stability for up to 43 days, with LEC1, in particular, showing a minimal reduction in the plaque concentration (Figure S6).

### 3.7. Efficacy of the Phage-Coated Container in Reducing E. coli Contamination on Fresh Vegetables

**Figure 7:**
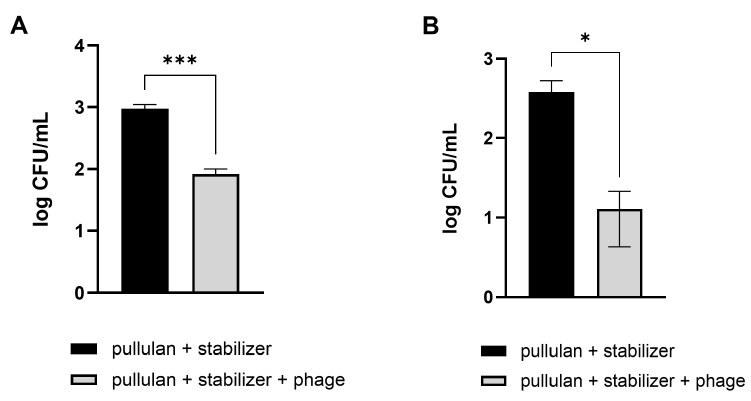
Efficacy of phage-coated rinsing containers in reducing *E. coli* O157:H7 levels in vegetables. Pathogenic *E. coli*-contaminated Brussels sprouts (**A**) and broccoli (**B**) were washed in an LEC1-coated container. After 10 min, the numbers of remnant *E. coli* on the vegetables were counted. Values represent the means with standard deviations of three trials. The marks above the bars, * or ***, represent significant differences at *p* < 0.05 or *p* < 0.001, respectively.

Vegetables that are difficult to wash, such as Brussels sprouts and broccoli, were used, similar to those used in the previous experiment. Pathogenic *E. coli* were artificially applied on the cut sections of Brussels sprouts and broccoli. Afterward, the contaminated vegetables were washed using a phage-coated rinsing container and water. A coating with pullulan and the stabilizer without phages was used as a negative control. As expected, vegetable washing using phage-coated containers was more effective in reducing the number of *E. coli*, showing 91% and 97% reductions in pathogenic *E. coli* numbers on Brussels sprouts and broccoli, respectively, compared to the levels observed after washing with tap water (Figure 7).

## 4. Discussion

During phage preservation, several factors can influence phage stability. The production process of phage formulations, compounds and ingredients of the preparations, forms of preparation, storage conditions, and route of phage application may induce variations in phage titers [33]. In addition, many environmental factors, such as temperature, pH, high hydrostatic pressure, the presence of surface-active compounds, and the ionic strength of the solution, affect phage stability [34,35,36]. The stability of phages is affected by environmental factors and morphological features [37]. Among different families and within one family, phage resistance to unfavorable physical and chemical factors varies. Phages with long tails and large and delicate virion structures are considered more sensitive to different kinds of stress [38,39]. In this study, two phages were used. Based on the comparison of genome sequence homology and microscopic observation results, we predicted that phage LEC1 belongs to the *Straboviridae* family and phage LSA5 to *Herelleviridae*. The instability and increased sensitivity of phage LSA5 may be attributed to the presence of longer tails that characterize the members of the *Herelleviridae* family. Additionally, the initial concentration of phages would potentially affect their stability. In a study by Hans et al. in 2021, four types of phages were prepared at two initial concentrations (9 and 7 log PFUs/mL) to assess stability over time [25]. They observed variations in stability, with all phages showing notably greater stability at 9 log PFUs/mL compared to 7 log PFUs/mL. In our study, for LEC1 and LSA5, stability experiments in different solutions (Figure 1) revealed that LEC1 started at an initial concentration of 8 log PFUs/mL, while LSA5 was at 7 log PFUs/mL, indicating a lower initial concentration for LSA5. This difference may have contributed to the lower stability observed for LSA5. However, in experiments evaluating the stabilizer’s efficacy (Figure 2), despite starting at low concentrations (7.7 log PFUs/mL for LEC1 and 6.5 log PFUs/mL for LSA5), both phages maintained their antibacterial activity for nearly a year, even when stored at room temperature following the addition of the stabilizer. This highlights the potential of the stabilizer to enhance phage stability, even under conditions where stability could be compromised.

Adding salts such as NaCl and MgSO_4_ considerably affected the persistence of stored phages in a liquid state. The following mechanism can explain the effect of the salts. Previous studies have reported that in solutions containing small amounts of salts (0–3 g/L of NaCl), phage MS2 showed a loss of infectivity of up to 90% and broken particles. In contrast, in solutions of high ionic strength with the addition of NaCl up to 5 g/L, phage MS2 retained its biological activity. The loss of infectivity was due to particle damage and aggregation. To demonstrate this, qRT-PCR was performed without the RNA extraction step, which detects free viral RNA from noninfectious phages, and only a small proportion was detected. This suggests that the loss of biological activity observed may be directly attributed to the breakup of the capsid, which results in RNA release [40]. In another study, a reduction in the titer of phage MS2 in a low ionic strength environment was attributed to phage aggregation, as evidenced by a size analysis of suspensions [41]. Szermer-Olearnik et al. demonstrated that a low concentration of Na^+^ ions (10 nM) led to the formation of a structure containing 20–100 phage virions (aggregation state) with a relatively low titer at 1.4 × 10^8^ PFUs/mL; however, a return to high ionic strength (150 nM) resulted in the reversal of phage particles to a dispersed state and the titer increased to 2.9 × 10^8^ PFUs/mL. The phage titer increase and structural recovery (dispersed state) from the aggregated structure were feasible with the increase in the Na^+^ ion concentration. The authors suggest that aggregation/dispersion of phages in different concentrations of salts does not reflect phage inactivity but is just a practical use of aggregates, such as in micro-filter formulations [42].

By adding salts, not only maintenance of phage titers but also other effects can be expected. Another study reported that a phage suspension retained its stability at 30 °C for over a month with added Mg^2+^ [43]. The incorporation of lithium, sodium, potassium, and magnesium cations can prevent phage inactivation not only at room temperature but also at high temperatures (50–70 °C) [44]. Even though the present study focused on the stability of phages at room temperature for convenient distribution and fresh food washing, these aqueous phage solutions can be applied under various conditions, including food processing steps at high temperatures.

In this study, adding sugars also assisted in maintaining phage titers during storage under unfavorable conditions. Generally, sugars protect proteins from a loss of activity due to chemical or thermal denaturation in solution. This effect is due to the increased surface tension of water, which raises the energy required to create cavities responsible for protein denaturation [45]. Sorbitol and mannitol, in particular, are widely used stabilizers that protect proteins from thermal denaturation [45,46]. Sugars are also known to have effects on protein unfolding [47]. Sugars inhibit the aggregation rate of viruses and enhance the stability of the secondary and tertiary structures of virus polypeptides, the loss of which is related to the concentration-dependent aggregation of viruses at high temperatures [48]. As such, adding sugars as stabilizers maintained phage activity by preventing morphological changes in the phage structure.

**Figure 8:**
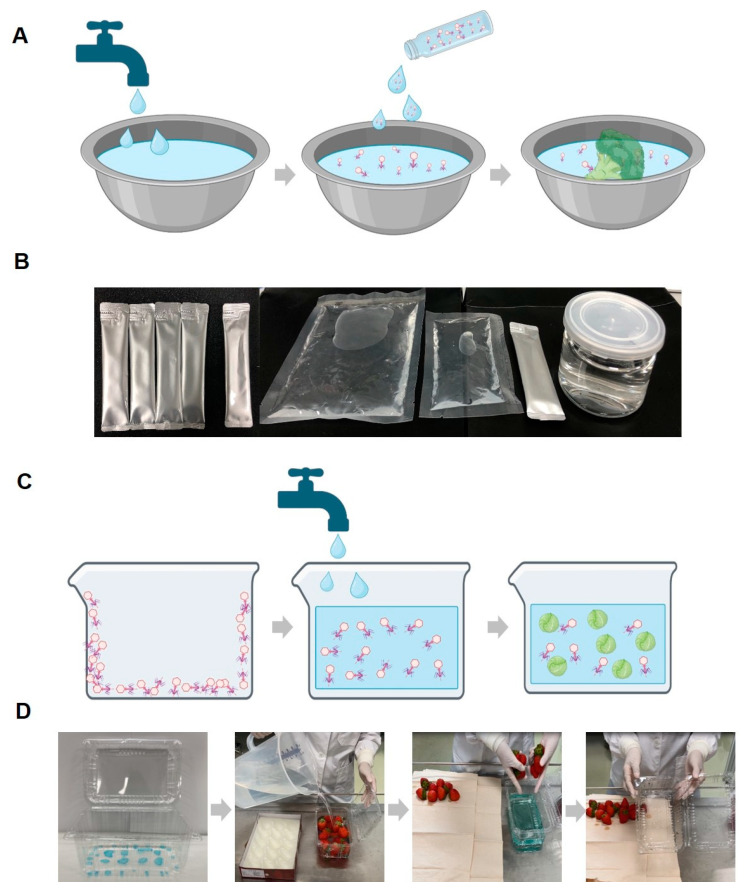
Schematic diagrams and example photos of the phage solution and coating application methods. Schematic diagram of the procedure for utilizing the phage solution (with stabilizer) in food washing (**A**) and example photo of the phage solution product forms (**B**). Schematic diagram of the washing method using phage-coated food containers (**C**) and example photos of a phage-coated container and the washing method using it (**D**). While the coating described in the manuscript is transparent, for experimental purposes, it was intentionally colored blue using edible dye to confirm the presence of the coating and its complete dissolution in water.

Based on these reasons, previous studies have continually attempted to enhance phage stability using salts or sugars in various forms (liquid, dried, and encapsulated) [25,49,50,51]. Furthermore, in our research, we established a final stabilizer condition by combining sugars and salts, demonstrating that the stabilizer can be applied in multiple forms. As a result, we provide examples of how stable phages can be applied to food in different forms during storage, effectively inhibiting pathogenic *E. coli* contamination (Figure 8).

The remarkable antibacterial activity of phages has been well-established. However, developing strategies to ensure long-lasting antibacterial activity becomes critical to explore their potential in various applications fully. In addition, the higher the level of convenience, the greater the value of its practical application. Regarding phage products for food applications, phage diffusion in the food matrix, especially in solid foods, is considerably limited. This may hinder the contact between phage and target bacteria or even provoke the inactivation of phage particles, resulting in differences in the efficacy of phages as biopreservatives, depending on the chemical composition, pH, and structure of the food matrix [52,53]. The development of aqueous solutions for phages can address these limitations. The aqueous solution of phages developed in this study shows high antibacterial efficiency during washing and comprises ingestible components, making it suitable for consumption with food. Furthermore, the phages can maintain their activity steadily throughout the distribution by coating the phages with a stabilizer on plastic containers used for vegetable storage and distribution. Consequently, harmful bacterial contamination of vegetables can be effectively controlled by adding water without transferring the vegetables into another container.

In conclusion, this study suggests an economical and efficient method for phage preservation without the need for complicated and expensive processes. Additionally, we successfully confirmed the antimicrobial activity of phages as an antibacterial washing agent and convenient rinsing container in reducing pathogenic bacteria that contaminate fresh vegetables. We demonstrate that the developed stabilizer increases the stability of phages, both in liquid and film states, thereby enhancing the potential of phage use and the applicability of phages to improve food safety.

## Data Availability

The data presented in this study are available on request.

## Supplementary Materials

The following supporting information can be downloaded at https://www.mdpi.com/article/10.3390/v16071155/s1: Figure S1. Morphological and genomic characteristics of phage LSA5; Figure S2. Lytic activities of phages in liquid medium; Figure S3. Effect of ion addition on phage stability; Figure S4. Effect of sugars on phage stability; Figure S5. Confirmation of the lytic activity of long-term preserved phage compared to phage lysate; and Figure S6. Stability of coated phages during storage in a refrigerator.
