# Supplementary material for: Development of Stabilizing Solution for Long-Term Storage of Bacteriophages at Room Temperature and Application to Control Foodborne Pathogens
Source: Viruses. 2024 Jul 17;16(7):1155. doi: 10.3390/v16071155 (PMC11281399; doi:10.3390/v16071155)
Supplement: Supplementary file 1 [file viruses-16-01155-s001.zip › viruses-3055587-supplementary.pdf]

Supplementary Material

Article

Development of stabilizing solution for long-term storage of bacteriophages at room temperature and application to control foodborne pathogens

Eo-Jin Kim [1,2], Min-Cheol Lim [1], Min-Ah Woo [1], Byoung Sik Kim [2] and Jeong-A Lim [1,*]

[1] Food Safety and Distribution Research Group, Korea Food Research Institute, Wanju-gun, 55365, Republic of Korea; k.eojin@kfri.re.kr (Eo-Jin Kim), mclim@kfri.re.kr (Min-Cheol Lim), mawoo@kfri.re.kr (Min-Ah Woo), and jeongalim@kfri.re.kr (Jeong-A Lim).

[2] Department of Food Science and Biotechnology, ELTEC College of Engineering, Ewha Womans University, Seoul, 03760, Republic of Korea; k.eojin@kfri.re.kr (Eo-Jin Kim) and b.kim@ewha.ac.kr (Byoung Sik Kim).

* Correspondence: jeongalim@kfri.re.kr; Tel.: 82-63-219-9366

A

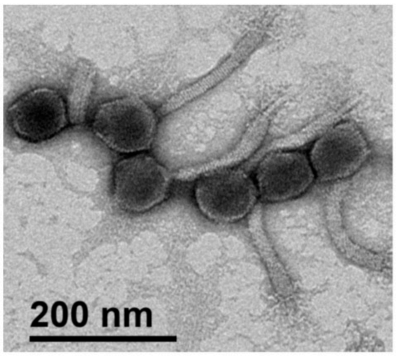

B

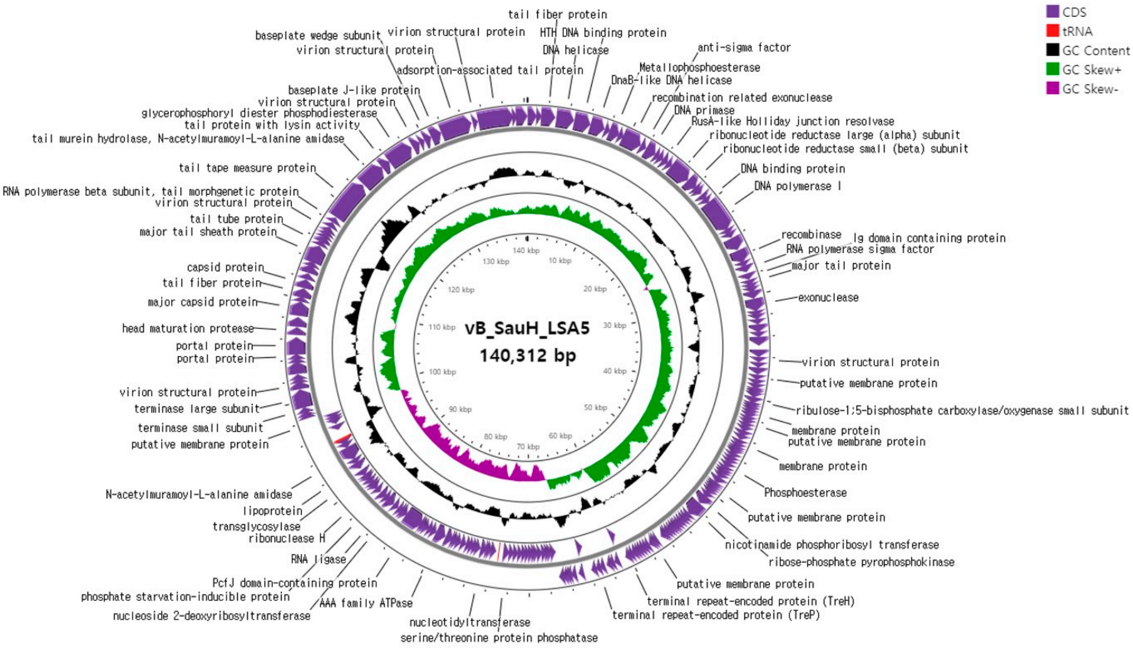

Figure S1. Morphological and genomic characteristics of phage LSA5. (A) The morphology of the phage was observed using a Transmission Electron Microscope (TEM). The phage was placed on a copper grid coated with a carbon nano film and negatively stained with 2% uranyl acetate solution. The stained samples were observed using a TEM (Hitachi 7650) operated at 100 kV. Observation of the morphological characteristics of phage LSA5 under the microscope revealed a head of approximately 70 nm and a tail of approximately 160 nm, confirming the presence of a baseplate structure. (B) The phage LSA5 genome consists of

140,312 bp with a GC content of 30.18%, containing 222 predicted genes and 4 tRNA genes. Specific functions could be predicted to 89 (40%) of the gene products. Gene products related to lysogeny, such as integrase, transposase, and repressor, as well as the genome attachment site, were not identified in the LSA5 genome, suggesting that it is a lytic phage. Furthermore, since no toxin or antibiotic resistance-encoding genes were identified, it suggests that LSA5 could be safely used as an antibacterial agent in food.

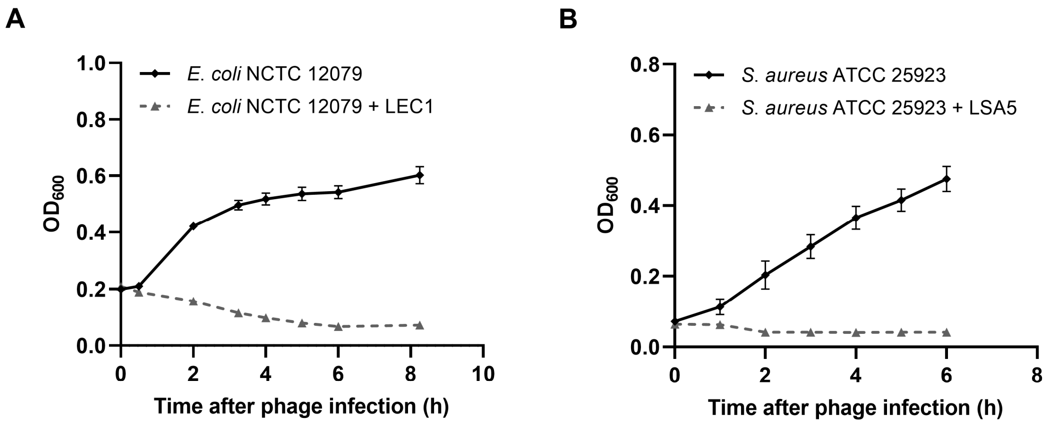

Figure S2. Lytic activities of phages in liquid medium. Each type of phage was added when the bacterial culture reached an early exponential phase. The solid lines in each graph indicate the growth of target bacteria without phage infection (negative control). The dotted lines indicate the growth of target bacteria infected with a phage. Values represent the mean with a standard deviation of three trials.

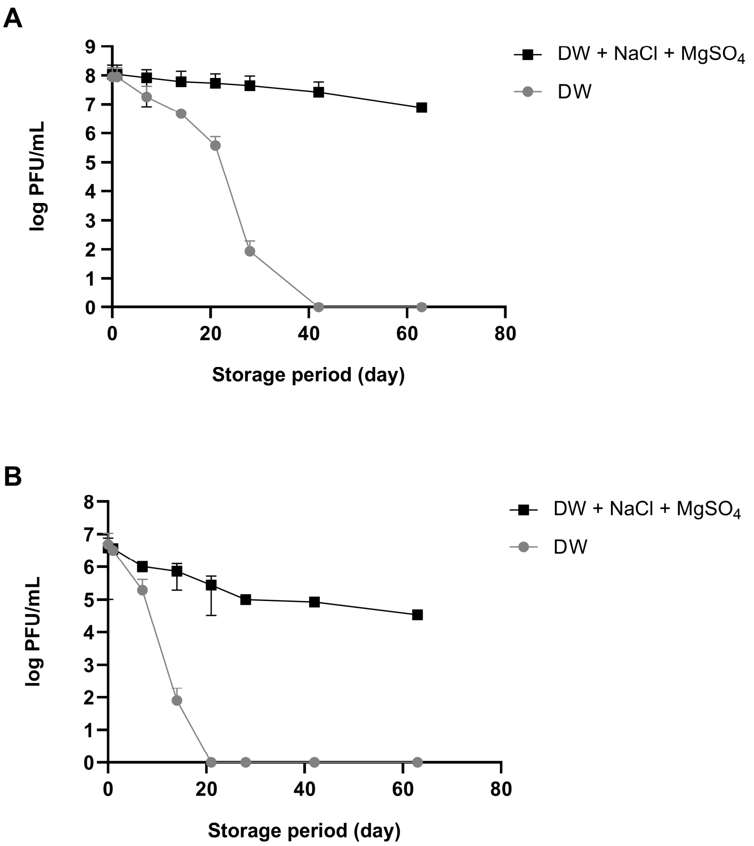

Figure S3. Effect of salt addition on phage stability. The phages were stored at room temperature in DW with or without adding 0.1 M NaCl and 8 mM $MgSO_4$. The plaques produced by phage in each solution were measured once every 1–2 weeks, on average, for up to 9 weeks. Values represent the mean with a standard deviation of three trials. (A) *E. coli* phage LEC1, (B) *S. aureus* phage LSA5.

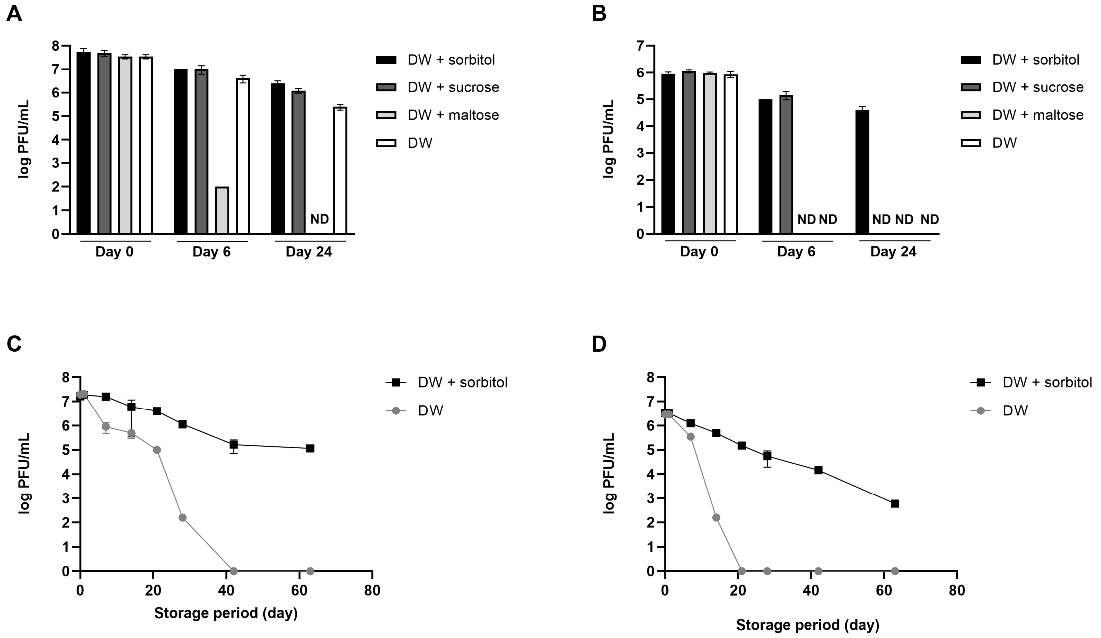

Figure S4. Effect of sugars on phage stability. Each sugar, sorbitol (black bar), sucrose (dark grey bar), or maltose (light grey bar), was added to DW to a final concentration of 10% (w/v). DW (white bar) was used as the negative control. The amount of phages LEC1 (A) and LSA5 (B) suspended in each solution was measured at 0, 6, or 24 days of storage. The plaque numbers of phages LEC1 (C) and LSA5 (D) stored in DW with or without 10% sorbitol were monitored during 9 weeks of storage at room temperature. Values represent the mean with a standard deviation of three trials. ND; not detected (limit of quantification; 10 PFU/mL).

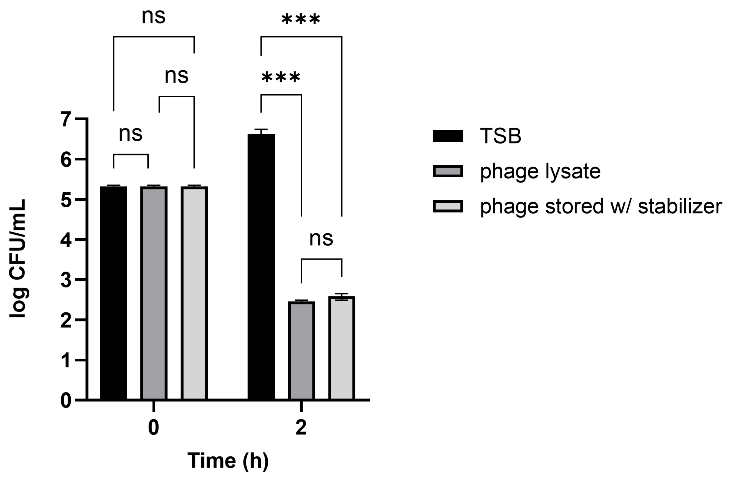

Figure S5. Confirmation of lysis activity of long-term preserved phage compared to phage lysate. A diluted *E. coli* culture was mixed with phage solution preserved with a stabilizer for 175 days at room temperature. As a positive control, phage lysate preserved in tryptic soy broth (TSB) in the refrigerator was used. TSB was used as the negative control. The numbers of viable *E. coli* were measured before and 2 h after phage addition. Values represent the mean with a standard deviation of three trials. *** Significant at $P < 0.001$. Ns: not significant.

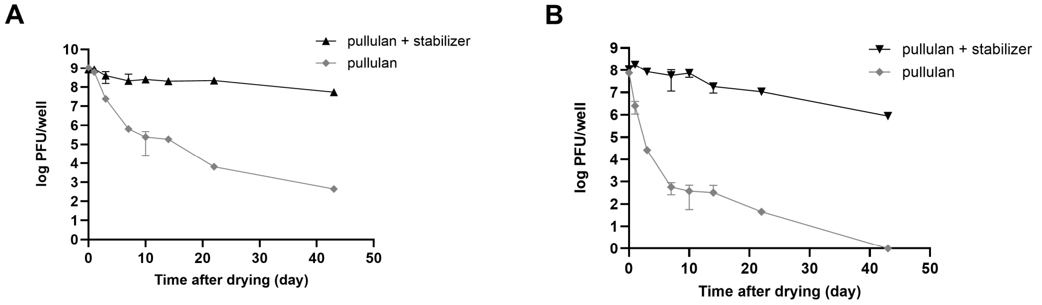

Figure S6. Stability of coated phages during storage at refrigerator. Phage LEC1 (A) and LSA5 (B) were mixed with pullulan w/ or w/o stabilizer and coated on the 6-well microplate. After drying, the plaque numbers of rehydrated phages were measured as a function of extended storage time. Values represent the mean with a standard deviation of three trials.
